# TSP50 promotes the Warburg effect and hepatocyte proliferation via regulating PKM2 acetylation

**DOI:** 10.1038/s41419-021-03782-w

**Published:** 2021-05-20

**Authors:** Feng Gao, Xiaojun Zhang, Shuyue Wang, Lihua Zheng, Ying Sun, Guannan Wang, Zhenbo Song, Yongli Bao

**Affiliations:** 1 National Engineering Laboratory for Druggable Gene and Protein Screening, Northeast Normal University, Changchun, China; 2 Research Center of Agriculture and Medicine Gene Engineering of Ministry of Education, Northeast Normal University, Changchun, China; 3 Key Laboratory of Molecular Epigenetics of the Ministry of Education, Northeast Normal University, Changchun, Jilin China

**Keywords:** Cancer metabolism, Oncogenes, Tumour biomarkers

## Abstract

Metabolic reprogramming is a hallmark of malignancy. Testes-specific protease 50 (TSP50), a newly identified oncogene, has been shown to play an important role in tumorigenesis. However, its role in tumor cell metabolism remains unclear. To investigate this issue, LC–MS/MS was employed to identify TSP50-binding proteins and pyruvate kinase M2 isoform (PKM2), a known key enzyme of aerobic glycolysis, was identified as a novel binding partner of TSP50. Further studies suggested that TSP50 promoted aerobic glycolysis in HCC cells by maintaining low pyruvate kinase activity of the PKM2. Mechanistically, TSP50 promoted the Warburg effect by increasing PKM2 K433 acetylation level and PKM2 acetylation site (K433R) mutation remarkably abrogated the TSP50-induced aerobic glycolysis, cell proliferation in vitro and tumor formation in vivo. Our findings indicate that TSP50-mediated low PKM2 pyruvate kinase activity is an important determinant for Warburg effect in HCC cells and provide a mechanistic link between TSP50 and tumor metabolism.

## Abstract (web summary)

Gao et al. shows that testes-specific protease 50 (TSP50) binds to PKM2 and promotes the Warburg effect by increasing PKM2 K433 acetylation level and PKM2 acetylation site (K433R) mutation remarkably abrogated the TSP50-induced aerobic glycolysis, cell proliferation in vitro and tumor formation in vivo. Our study reveals a link between an oncogene and a key enzyme in HCC glycolysis, which provides new ideas for human HCCs treatment with TSP50 as the target.

## Graphical abstract

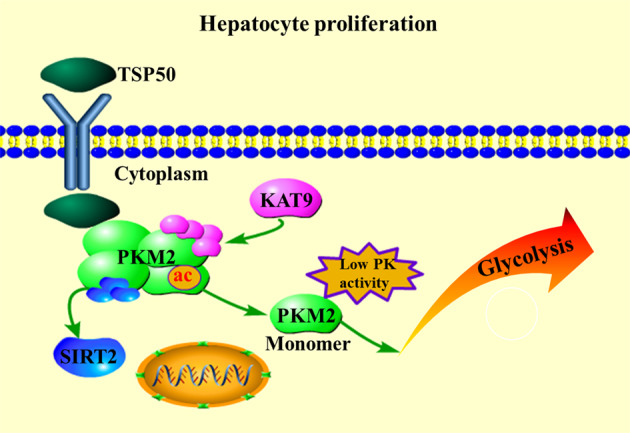

## Introduction

In the presence of sufficient levels of oxygen, normal quiescent cells metabolize glucose to pyruvate, which is further oxidized through oxidative phosphorylation to generate ATP for cellular processes while more glucose is metabolized into lactate under hypoxic conditions. However, even in the presence of sufficient oxygen, tumor cells can reconstruct the process of glucose metabolism, limiting energy metabolism largely to glycolysis, which is called aerobic glycolysis or Warburg effect^1,2^. This cell metabolism reprogramming allows tumor cells to maintain the balance between energy demand and synthetic metabolism, which is conducive to the synthesis of biomacromolecules to promote cell proliferation^3^. The increase of aerobic glycolysis is a widely observed feature in human cancers and often correlates with tumor invasiveness and poor prognosis of human hepatocellular carcinoma (HCC)^4–6^.

The activation of oncogenes plays an important role in driving the development of tumor metabolism for improving the adaptability of tumor cells and promoting cells proliferation and survival^7–11^. Importantly, interfering with the metabolic changes of tumor cells reduces tumorigenicity and increases apoptosis sensitivity to chemotherapeutic drugs, indicating that aerobic glycolysis metabolism is a key metabolic factor for tumor development^12–15^. Thus, a better understanding of the mechanistic links between cellular metabolism and regulatory genes can be great significance for the development of new treatments, especially in HCC, considering the close correlation between metabolic changes and the pathogenesis of HCC. In the physiological state, TSP50 is specifically expressed in spermatocytes of testis, while high expression of TSP50 can be detected in more than 90% of breast cancer, laryngeal cancer, colorectal cancer, cervical cancer and gastric cancer tissue samples^16–20^, suggesting that TSP50 is of great significance in the diagnosis of tumors. After knocking down TSP50 in mouse teratoma cell line P19 and laryngeal cancer cell Hep2, the ability of cell proliferation and colony formation are decreased significantly^21^, and obvious apoptosis occurs^17^. In addition, the natural compounds Alantolactone and Cardamom have been found to significantly reduce the TSP50 level, thereby inhibiting the gastric cancer cells and breast cancer cells proliferation and inducing their apoptosis^22^. We have recently shown that TSP50 can promote CHO cells and MCF-10A cells proliferation, clonal formation and tumorigenic ability and TSP50 knocking-down exerts an inhibitory effect on the migration and invasion of MDA-MB-231 and MDA-MB-435S cells^23^. Meanwhile, the function of TSP50 in promoting invasion and metastasis is mainly achieved by increasing the expression of NF-κB-dependent MM9^23^. Further studies have shown that IκBα, a key signal molecule of NF-κB signal pathway, is degraded after binding to TSP50, thus promoting cell proliferation and inducing cell transformation^24^. The threonine protease activity and catalytic triad of TSP50 are essential for its function in cell proliferation^25,26^. In breast cancer cells, TSP50 can also promote cell proliferation partly by inhibiting activin signal transduction^16^. These findings suggest that TSP50 plays an important role in the diagnosis, prognosis and treatment of tumors. However, whether the proliferation-promoting function of TSP50 is partly due to the increased Warburg effect in human cancer cells has not been elucidated before.

PKM2 is recognized as the key rate-limiting enzyme that regulates aerobic glycolysis in tumor cells, catalyzing the synthesis of pyruvate and ATP using phosphoenolpyruvate and ADP^27,28^. PKM1 and PKM2 are encoded by the PKM gene. PKM1 is mainly expressed in muscle or brain tissues that need a large amount of energy supply, whereas PKM2 is significantly expressed in rapidly proliferating tissues such as embryonic cells, stem cells or tumor cells^29,30^. PKM2 in tumor cells, but not its spliced variant PKM1, is often depolymerized into low-activity dimers or inactive monomers, resulting in a decrease of PK activity that promotes the Warburg effect and favors cancer cell growth and survival^31–33^. On the contrary, the high PK activity of PKM2 exerts an inhibitory effect^33–35^, emphasizing the importance of identifying endogenous regulators of PKM2 activity in cancer.

In this study, we found that TSP50 was highly expressed in a variety of HCC cells. TSP50 could directly bind to PKM2 to positively regulate aerobic glycolysis through the influence of PKM2 pyruvate kinase activity, thus promoting the survival of HCC cells. We demonstrated that TSP50 maintained the low pyruvate kinase activity of PKM2 by directly mediating the acetylation of PKM2 at K433. In summary, we reveal a molecular link between cell survival regulators and key enzymes of cancer metabolism and our study will provide potential therapeutic significance for HCC patients.

## Results

### TSP50 is highly expressed in HCC and survival-associated

**Fig. 1:**
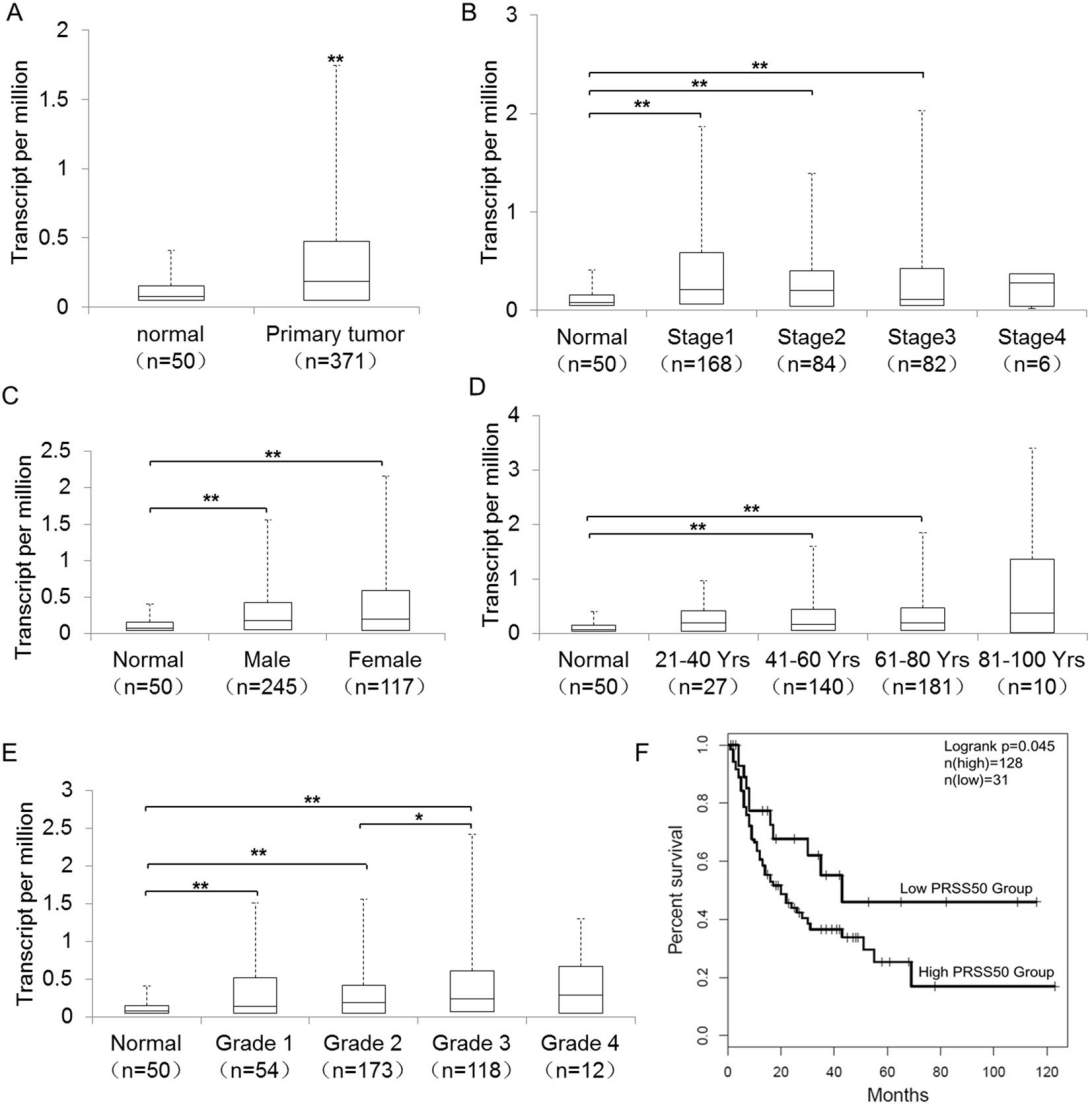
TSP50 is elevated in HCC and survival-associated. **A** Boxplot showing relative expression of TSP50 in normal and HCC samples. **B** Boxplot showing the relative expression of TSP50 in normal individuals and HCC patients in different stages. **C** Boxplot showing the TSP50 relative expression in normal individuals as well as male and female HCC patients, respectively. D Boxplot showing relative expression of TSP50 in normal individuals of any age and LIHC patients aged 21–40, 41–60, 61–80, or 81–100 yr. **E** Boxplot showing the TSP50 relative expression in normal individuals and HCC patients with grade 1, 2, 3 or 4 tumors. **F** GEPIA analysis of the correlation between TSP50 mRNA expression levels and RFS of HCC patients. **A**–**E** UALCAN database. **F** GEPIA database. The Student’s *t*-test was used to estimate the significance of difference between two groups and more than two groups were analyzed by one-way ANOVA. *P < *0.05*, ** P < *0.01*.

We evaluated TSP50 transcription levels via UALCAN database^36^. Analysis results revealed that mRNA expression of TSP50 was significantly higher in TCGA-LIHC tissues than in adjacent normal tissues (Fig. 1A). Further sub-group analysis of multiple clinic-pathological features of TCGA-LIHC samples based on disease stages, gender, age, and tumor grade showed that the expression of TSP50 was significantly higher in HCC patients than normal controls (Fig. 1B–E). The comparisons results made within different tumor stages and grades showed that the expression level of TSP50 in tumor grade 2 subgroup was significantly lower than that in grade 3 group, however, there was no significant difference among other subgroups, suggesting that TSP50 expression was not stage or grade dependent (Fig. 1B and E). Then, GEPIA was employed to generate survival curves (recurrence-free survival,RFS) to assess the association between TSP50 expression and the survival outcomes of HCC cohorts^37^. The HCC patients were separated into two groups according to the TSP50 mRNA expression level. Finally, we found that the high TSP50 expression group had a shorter RFS (Fig. 1F). Thus, TSP50 expression may serve as a potential diagnostic indicator in HCC.

### TSP50 promotes cell proliferation and drives glycolytic metabolism in HCC cells

**Fig. 2:**
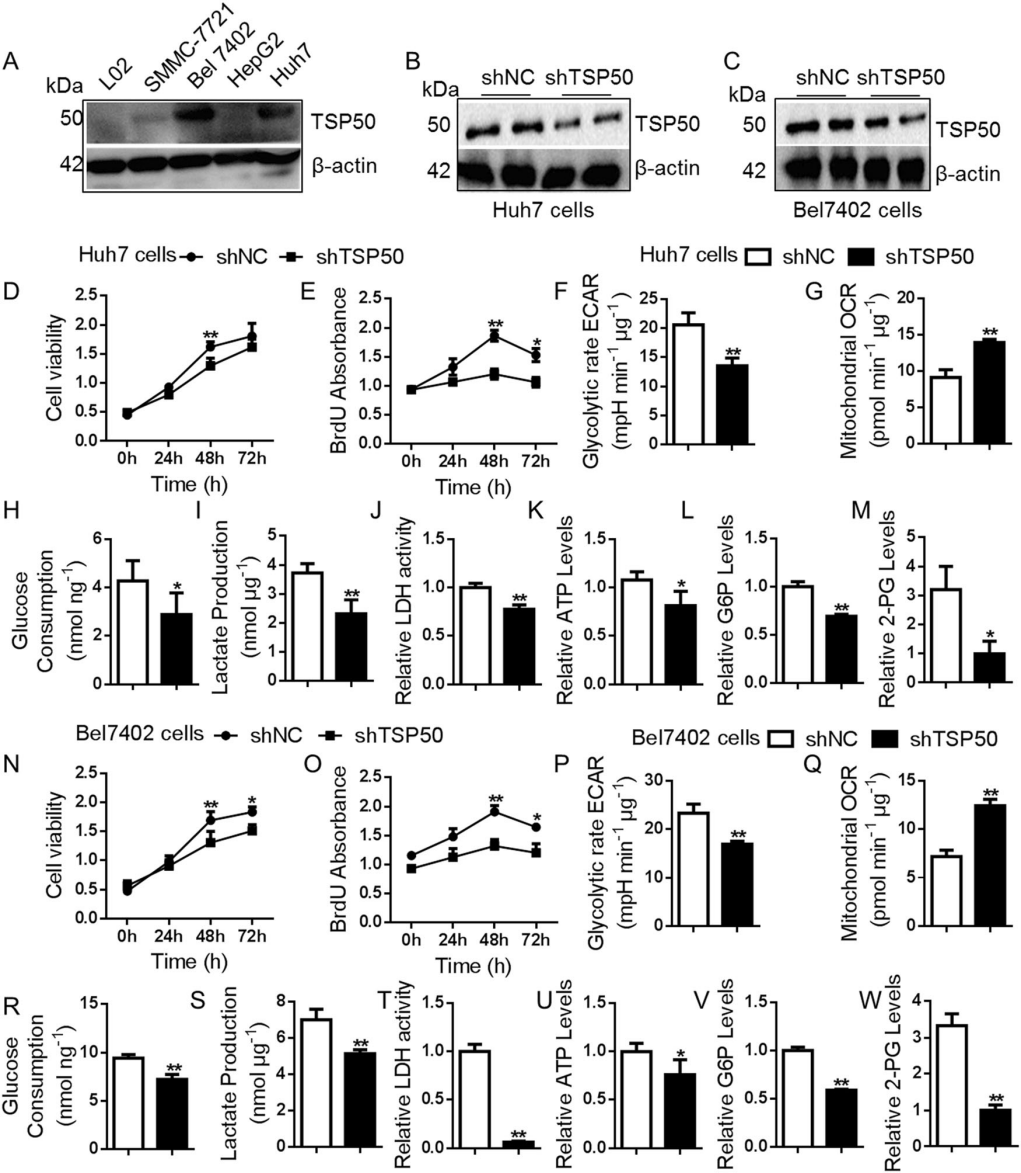
shTSP50 affects cell proliferation and aerobic glycolysis. **A** Multiple cancer cells were cultured, harvested and subjected to Western blot analysis for TSP50 detection. **B**–**C** shTSP50 was transfected into Huh7 and Bel7402 cells for the gene knockdown and the knockdown efficiency was evaluated. β-actin served as loading control. **D**, **E** The Huh7 cell viability and BrdU absorbance were detected in cells after shTSP50 transfection by MTT and BrdU assay. **F**–**M** After shTSP50 transfected in Huh7 cells, the ECAR, OCR, glucose consumption, lactate production, LDH activity, ATP levels, G6P levels and 2-PG levels were analyzed. **N**, **O** The Bel7402 cell viability and BrdU absorbance were detected in cells after shTSP50 transfection by MTT and BrdU assay. **P**–**W** After shTSP50 transfected in Bel7402 cells, the ECAR, OCR, glucose consumption, lactate production, LDH activity, ATP levels, G6P levels and 2-PG levels were analyzed. The aerobic glycolysis values were normalized to protein levels. *N* = 3 biologically independent replicates. Data were presented as means ± s.d. *P < 0.05, **P < 0.01 as compared to NC group by two-sided Student’s *t*-test. ns, no significance.

**Fig. 3:**
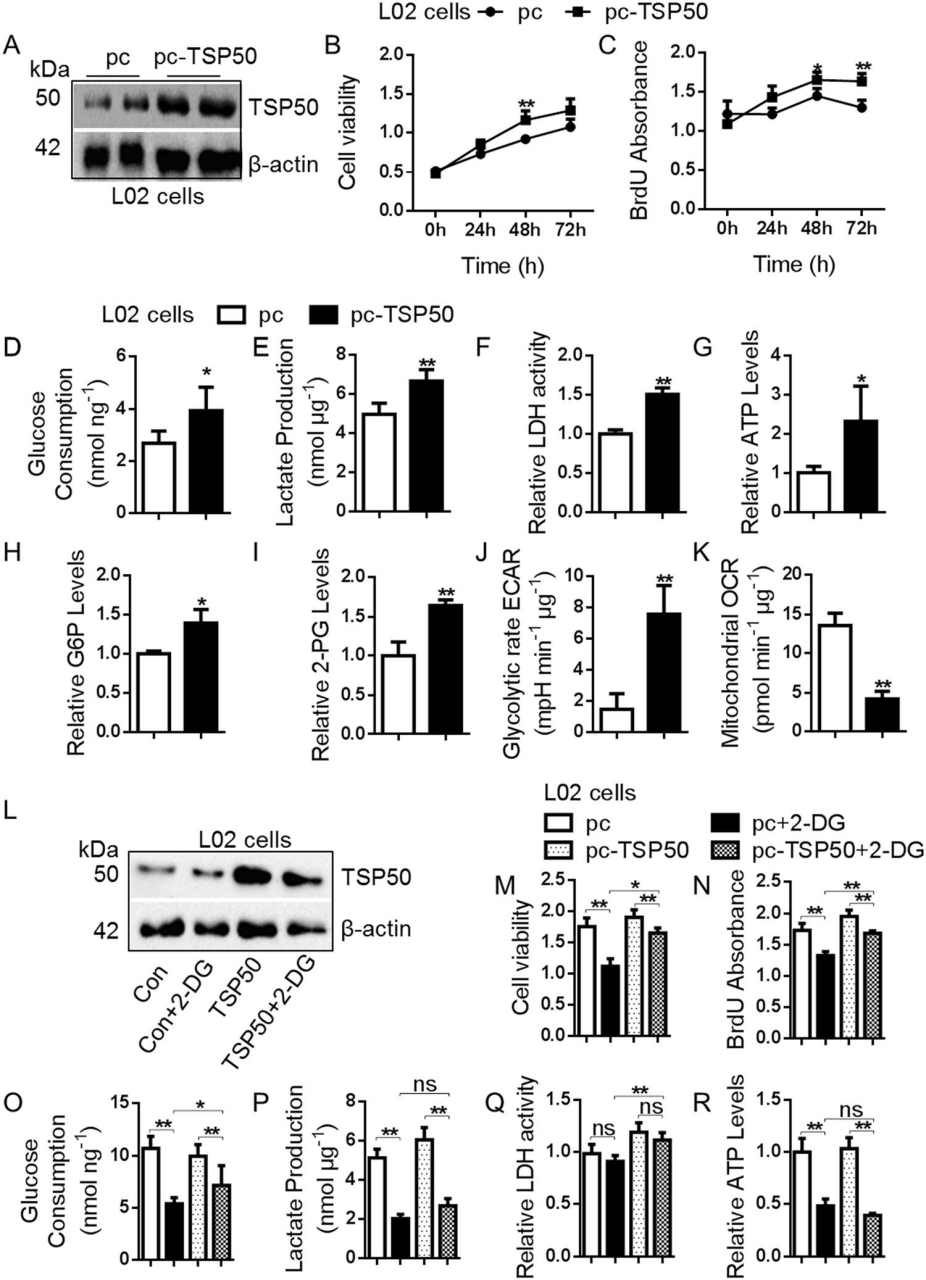
Over-expressed TSP50 affects cell proliferation and aerobic glycolysis. **A** pcDNA3.1-TSP50 was transfected into L02 cell to overexpress TSP50 and efficient expression of TSP50 was examined by Western blot. **B**, **C** The L02 cell viability and BrdU absorbance were detected in cells after pcDNA3.1-TSP50 transfection by MTT and BrdU assay. **D**–**K** After pcDNA3.1-TSP50 transfected in L02 cells, the ECAR, OCR, glucose consumption, lactate production, LDH activity, ATP levels, G6P levels and 2-PG levels were analyzed. **L** Effect of 2-DG on TSP50-overexpressed L02 cells, the expression of TSP50 was examined by Western blot. **M**, **N** The cell viability and BrdU absorbance were detected by MTT and BrdU assay. **O**–**R** glucose consumption, lactate production, LDH activity and ATP levels were analyzed. The aerobic glycolysis values were normalized to protein levels. *N* = 3 biologically independent replicates. *t*-Test or one-way ANOVA statistical analysis were used. Data were presented as means ± s.d. *P < 0.05, ** P < 0.01. ns, no significance.

The expression of TSP50 in different cell lines (L02 cells, SMMC-7721 cells, Huh7 cells, HepG2 cells and Bel7402 cells) was detected. Western blot results showed that TSP50 was almost not detected in normal L02 cells, whereas higher expressed TSP50 was found in Huh7 and Bel7402 HCC cells (Fig. 2A). To investigate the effect of TSP50 on cell proliferation, Huh7 and Bel7402 cells were transfected with shTSP50 plasmids for TSP50 knockdown (Fig. 2B, C). MTT and BrdU results showed that TSP50 knockdown significantly inhibited Huh7 and Bel7402 cell viability and proliferation (Fig. 2D–E, N–O). Studies have indicated that tumor cells underwent metabolic reprogramming to adapt to rapid proliferation and enhanced the aerobic glycolysis pathway^38^. To figure out whether alteration of TSP50 directly influence glycolytic metabolism, we measured extracellular acidification rate (ECAR), oxygen consumption rate (OCR), glucose consumption, lactate production, LDH activity, ATP, G6P and 2-PG levels in HCC cells after TSP50 knockdown. The results showed that TSP50-knockdown HCC cells revealed lower ECAR levels and higher OCR levels, meanwhile, other aerobic glycolysis related indicators levels were also significantly reduced (Supplementary Fig. S1, Fig. 2F–M, P–W). Subsequently, we overexpressed TSP50 with pcDNA3.1-TSP50, which induced the overproliferation of L02 cells (Fig. 3A–C). Further functional colorimetric validation showed that TSP50 increased glucose consumption, lactate production, LDH activity, ATP as well as G6P and 2PG levels (Fig. 3D–I). Meanwhile, TSP50 overexpression increased ECAR levels and reduced OCR levels in L02 cells compared with control cells (Supplementary Fig. S1, Fig. 3J, K). After obtaining the above results, we further examined the effect of TSP50 on the levels of regulatory factors in the glycolytic pathway. We found that TSP50 could promote the expression of glycolysis-related proteins such as GLUT1, HK2, PKM2, etc (Supplementary Fig. S2).

In addition, 2-deoxy-d-glucose (2-DG) was applied to TSP50-overexpressing L02 cells to block glycolysis and we observed that the levels of aerobic glycolysis and the proliferation ability of L02 cells were significantly decreased. In detail, the ability of aerobic glycolysis and cell proliferation in TSP50 + 2-DG group were significantly higher than 2-DG group and lower than those in the TSP50 overexpression group (Supplementary Fig. S3, Fig. 3L–R). Taken together, our data suggested that TSP50 was beneficial to cell proliferation and aerobic glycolysis, and the tumor-promoting effect of TSP50 was partly dependent on aerobic glycolysis.

### TSP50 interacts with PKM2 and depends on PKM2 for its metabolic effects

**Fig. 4:**
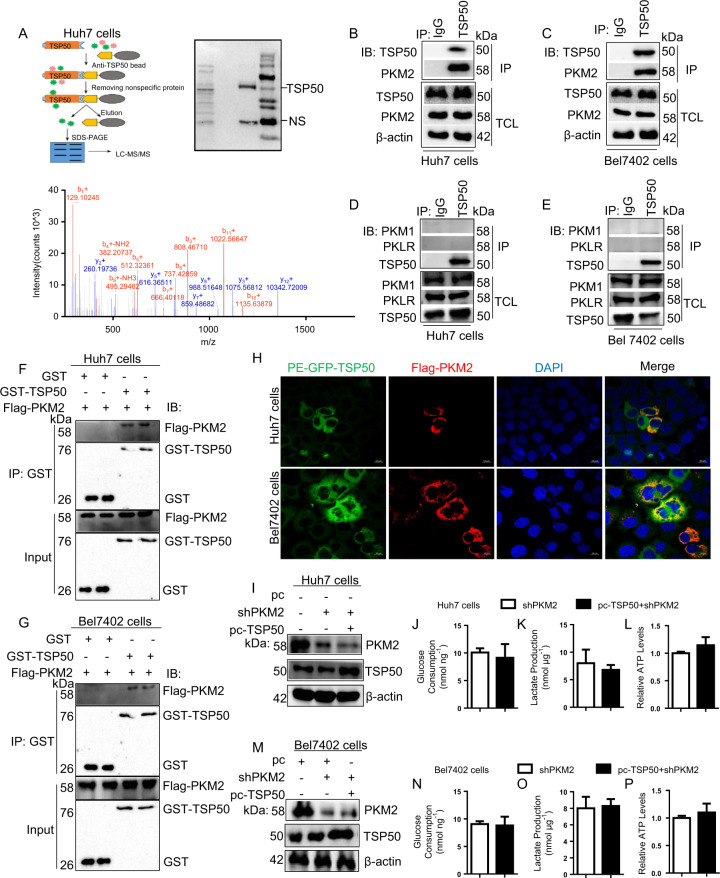
TSP50 binds with PKM2 and depends on PKM2 for its metabolic effects. **A** Identification of proteins binding to endogenous TSP50 by LC-MS/MS in Huh7 cells. Candidate binding proteins to TSP50 were obtained after non-specific proteins were removed. **B**, **C** Huh7 and Bel7402 cells were harvested and subjected to immunoprecipitation with anti-TSP50 antibody, followed by Western blot analysis with anti-PKM2 antibody. **D**, **E** Huh7 and Bel7402 cells were harvested and subjected to immunoprecipitation with anti-TSP50 antibody, followed by Western blot analysis with anti-PKM1 or anti-PKLR antibody. **F**, **G** GST pull-down of Flag-PKM2 by GST-TSP50 using proteins purified in B21 bacteria, followed by Western blot analysis with anti-PKM2 and anti-GST antibody. **H** Huh7 and Bel7402 cells were fixed and subjected to immunofluorescence analysis. TSP50 co-localized with PKM2 in Huh7 and Bel7402 cells. Scale bar, 10 μm. **I**–**P** Glucose consumption, lactate production and ATP levels were analyzed after silencing of PKM2 abrogates the metabolic effects of TSP50. All aerobic glycolysis values were normalized to protein levels. *N* = 3 biologically independent replicates. *t*-Test statistical analysis was used. Data were presented as means ± s.d.

As TSP50 could apparently promote aerobic glycolysis, we infer that TSP50 may bind to metabolic enzymes, thereby regulating aerobic glycolysis. To identify the underlying target molecules regulated by TSP50, LC–MS/MS was performed and the results showed that PKM was one of the proteins interacting with TSP50 (Fig. 4A, data not shown). We verified this result with co-IP assay, which showed that endogenous TSP50 protein interacted with PKM2 protein in the cytoplasm rather than PKM1 or PKL/R (Fig. 4B–E). Recent studies have shown that PKM2 is a major regulator for metabolic reprogramming in cancer which contributes to the Warburg effect^28,39,40^. To further validate the interaction between TSP50 and PKM2 in HCC cells, GST pull-down and immunofluorescence assays were performed. We found that GST-tagged TSP50 protein could pull-down Flag-tagged PKM2 protein by Western blotting upon GST pull-down assays (Fig. 4F, G). As confirmed by Immunofluorescence, TSP50 and PKM2 were co-localized in the cytoplasm after GFP-TSP50 and Flag-PKM2 plasmids were co-transfected into Huh7 and Bel7402 cells (Fig. 4H). All the results above indicated that there was a close direct interaction between TSP50 and PKM2 proteins.

Further results showed that the effect of TSP50 on aerobic glycolysis level was significantly reduced in PKM2-knockdown Huh7 and Bel7402 cells (Fig. 4I–P), which confirmed that TSP50 was dependent on PKM2 for its metabolic effects.

### TSP50-mediated acetylation is essential for maintaining low level of tetrameric PKM2

**Fig. 5:**
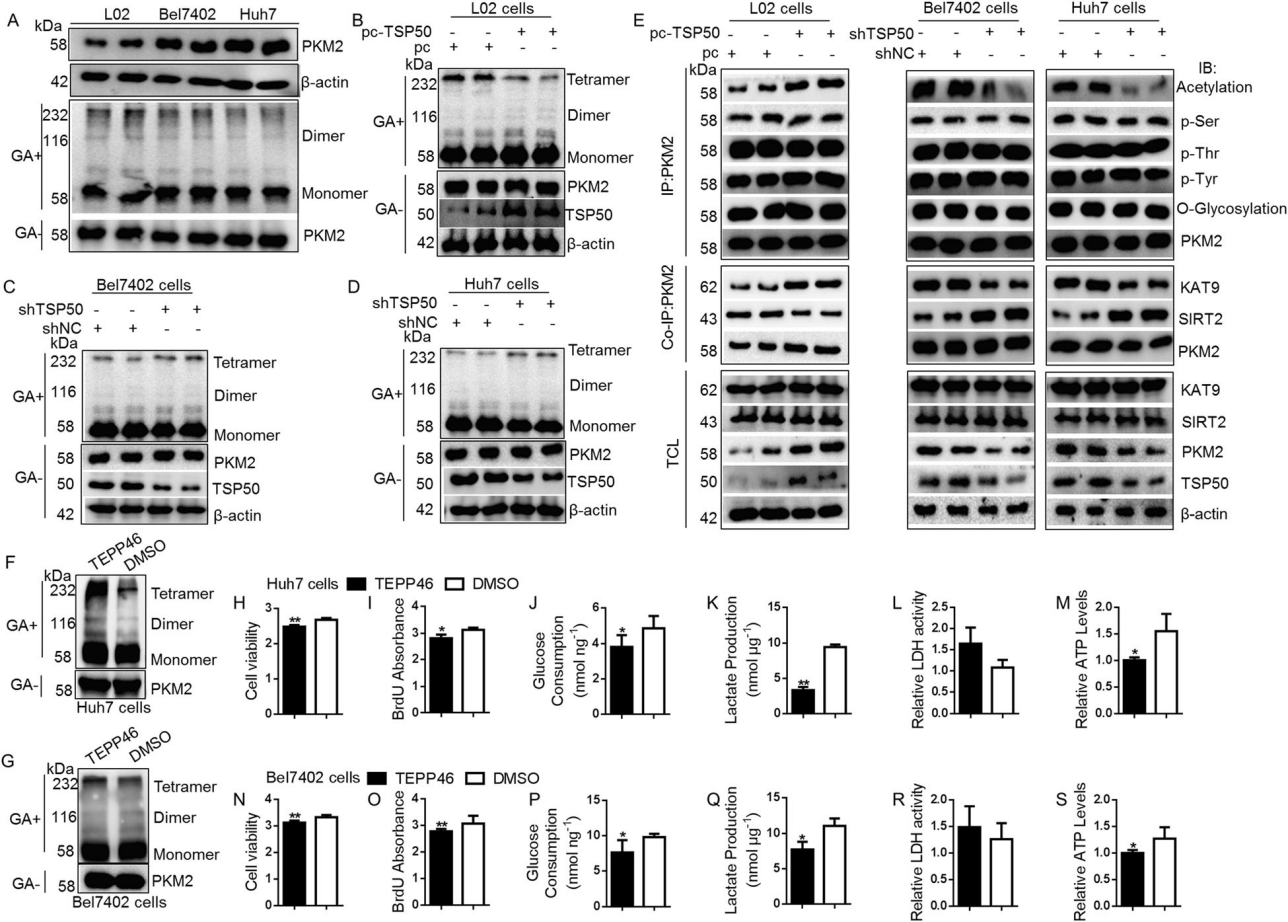
TSP50-mediated acetylation is essential for maintaining low level of tetrameric PKM2. **A** Comparison of PKM2 oligomer states in different cells. After extracting the cell protein and treating with glutaraldehyde, immunoblotting was performed with anti-PKM2 for oligomer states. **B**–**D** PKM2 oligomer states were detected in TSP50-overexpressed L02 cells and TSP50-knockdowned HCC cells. **E** TSP50 was overexpressed in L02 cells and knocked down in Huh7 cells and Bel7402 cells. Then, cells were harvested and followed by IP with anti-PKM2, Western blot analysis was performed with anti-PKM2, Acetylation, p-Tyr, p-Thr p-Ser and O-Glycosylation. Interaction of PKM2 with KAT9 and SIRT2 were analyzed by co-IP method. **F**–**S** After TEPP46 treatment, the cell viability, BrdU absorbance, glucose consumption, lactate production, LDH activity and ATP levels were analyzed. All aerobic glycolysis values were normalized to protein levels. *N* = 3 biologically independent replicates. *t*-Test statistical analysis was used. Data were presented as means ± s.d. *P < 0.05, ** P < 0.01.

In cancer cells, a constitutively low PKM2 activity is essential for aerobic glycolysis^31–33^. The oligomers of PKM2 exist in high activity tetramer, low activity dimer and inactive monomer forms. Therefore, we investigated a possible relationship between PKM2 and TSP50. We first compared the oligomeric state of PKM2 in different cells, and the results showed that, compared with L02 cells, the high-activity tetrameric form in Huh7 cells and Bel7402 cells was reduced (Fig. 5A). Furthermore, TSP50 overexpression in L02 cells led to a low level of PKM2 tetrameric form, while TSP50 knockdown in Huh7 and Bel7402 cells significantly increased tetrameric PKM2 (Fig. 5B–D).

The activity of PKM2 is regulated by various covalent modifications, such as phosphorylation^41,42^, acetylation^43,44^ and glycosylation^45^. We used immunoprecipitation assay to examine the effect of TSP50 on PKM2 protein modification. The results showed that TSP50 promoted the acetylation level of PKM2, with negligible effects on phosphorylation and glycosylation (Fig. 5E). Since TSP50 only has the activity of threonine hydrolase, we speculate that TSP50 may affect the binding ability of PKM2 to acetylase or deacetylase. Further detection results showed that TSP50 could reduce deacetylase SIRT2 level and increase acetylase KAT9 level, two proteins that interacted with PKM2 (Fig. 5E). Furthermore, TEPP-46, a PKM2 activator, was selected to detect the effect of altered PKM2 activity on cell proliferation and aerobic glycolysis levels. The results showed that TEPP 46 effectively activated PKM2 in HUH7 and BEL7402 cells (Fig. 5F–G). The proliferation and aerobic glycolysis levels of tumor cells treated with TEPP 46 were significantly lower than the control group cells (Fig. 5H–S). These results indicated that TSP50 inhibited PKM2 pyruvate kinase activity by mediating acetylation of PKM2 thereby promoting aerobic glycolysis and cell proliferation levels.

### PKM2 is acetylated at K433

**Fig. 6:**
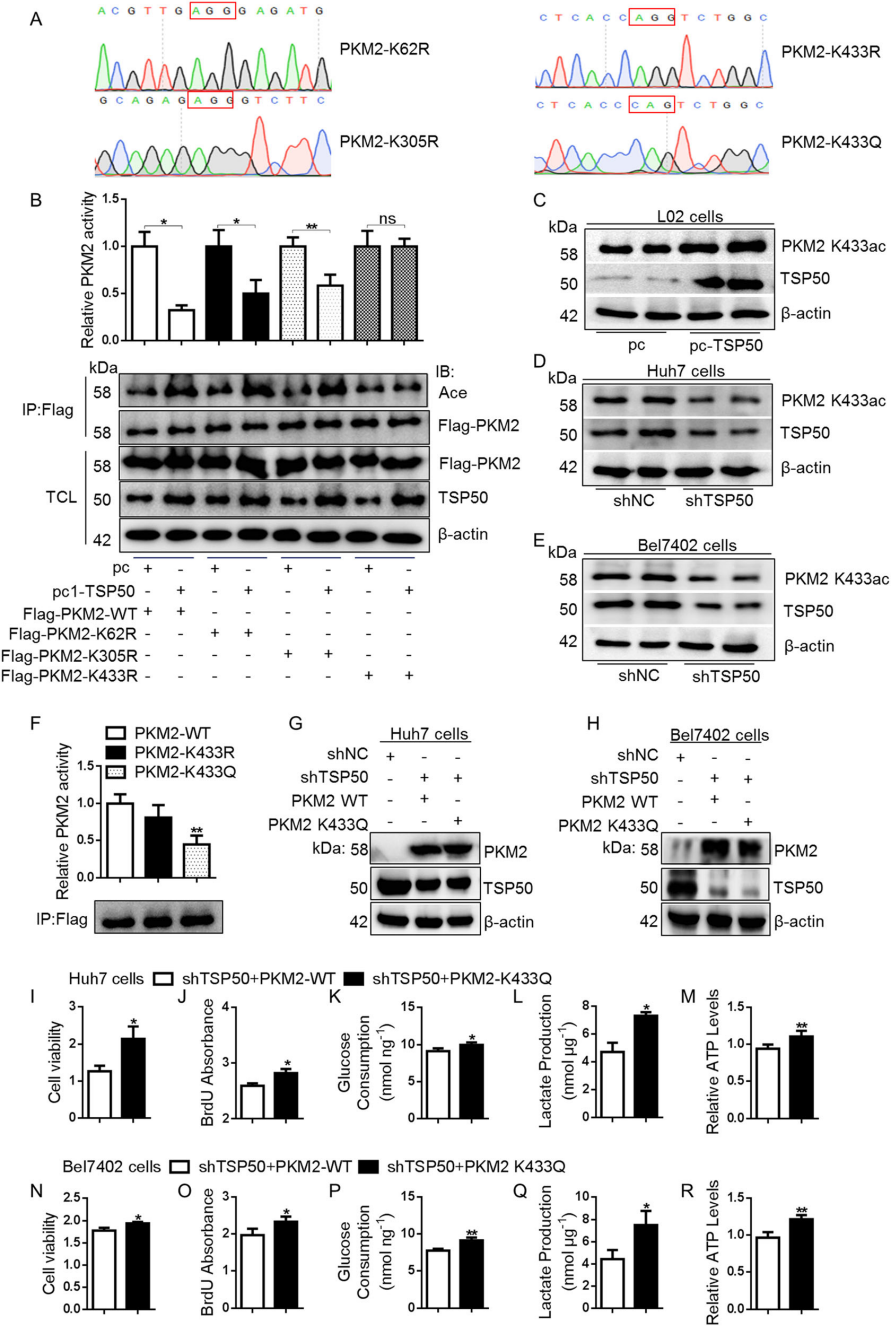
TSP50 acetylated PKM2 at K433 site. **A** Vectors encoding for multiple mutant PKM2 were constructed for subsequent analysis using a site-directed mutagenesis kit. **B** PKM2 K62R, K305R or K433R mutant vector was co-transfected with TSP50 into Huh7 cells. The cell was harvested and followed by IP with anti-Flag and PKM2 activity analysis. **C**–**E** The pcDNA3.1-TSP50 and Flag-PKM2 WT, Flag-PKM2K62R, Flag-PKM2 K305R or Flag-PKM2 K433R mutant plasmids were co-transfected into cells, respectively. The level of PKM2 K433 acetylation was detected using an anti-PKM2 K433ac antibody. **F**–**R** Acetylated mimic of PKM2 (PKM2-K433Q) transfected in cell lines deficient of TSP50, the PKM2 activity, cell viability, BrdU absorbance, glucose consumption, lactate production and ATP levels were detected. All aerobic glycolysis values were normalized to protein levels. *N* = 3 biologically independent replicates. *t*-Test statistical analysis was used. Data were presented as means ± s.d. *P < 0.05, **P < 0.01. ns, no significance.

We next sought to identify the PKM2 acetylation site(s) affected by TSP50. The acetylation sites of PKM2 are usually K62 lysine, K305 lysine and K433 lysine^44,46,47^. Therefore, the mutant plasmids of Flag-PKM2-K62R, K305R and K433R were constructed and transfected into Huh7 cells (Fig. 6A). Subsequently, after transfecting TSP50 in PKM2-WT, K62R, K305R and K433R cells, different forms of PKM2 protein were purified by immunoprecipitation, and the acetylation levels were detected. The results showed that PKM2 acetylation levels and PKM2 pyruvate kinase activity in PKM2 K433R cells were not affected by TSP50 (Fig. 6B). Finally, Western blot detection results with anti-PKM2 K433ac antibody showed that TSP50 could promote the acetylation level of PKM2 K433 site (Fig. 6C–E). Together, these results demonstrated that TSP50 acetylated PKM2 at K433.

### TSP50 mediates the cell proliferation and aerobic glycolysis changes partially dependent on PKM2 K433 acetylation

Studies have shown that pyruvate kinase mutations affecting PKM2 activity and thus cancer metabolism^48^. The PKM2 pyruvate kinase activity is significantly lower when PKM2 lysine 305 is substituted with glutamine (K305Q)^35,44,46^. In contrast, PKM2 K62Q mutation has no effect on PKM2 pyruvate kinase activity^44,46^. The effect of PKM2 K433Q on PKM2 pyruvate kinase activity has not been studied, however, it has been shown that the increase of PKM2 K433 acetylation level can reduce the accumulation of PKM2 tetramer, leading to a decreased PKM2 activity^49,50^. In this study, to determine how PKM2 K433 acetylation affects PKM2 activity, we constructed a Flag-PKM2-K433Q mutant plasmid (Fig. 6A). The PKM2 WT and PKM2 K433Q mutants were immunopurified from the transfected HEK-293T cells and PKM2 activity was measured. We found that the PKM2 pyruvate kinase activity of PKM2 K433Q mutant was significantly reduced (Fig. 6F). Meanwhile, in TSP50-knockdown Huh7 and Bel7402 cells, when PKM2 lysine 433 was substituted with glutamine (K433Q), cell proliferation and aerobic glycolysis level were increased significantly, as compared to PKM2 WT-transfected control cells (Fig. 6G–R). Together, these results demonstrated a strong effect of high K433 acetylation level in inhibiting PKM2 pyruvate kinase activity and promoting aerobic glycolysis.

**Fig. 7:**
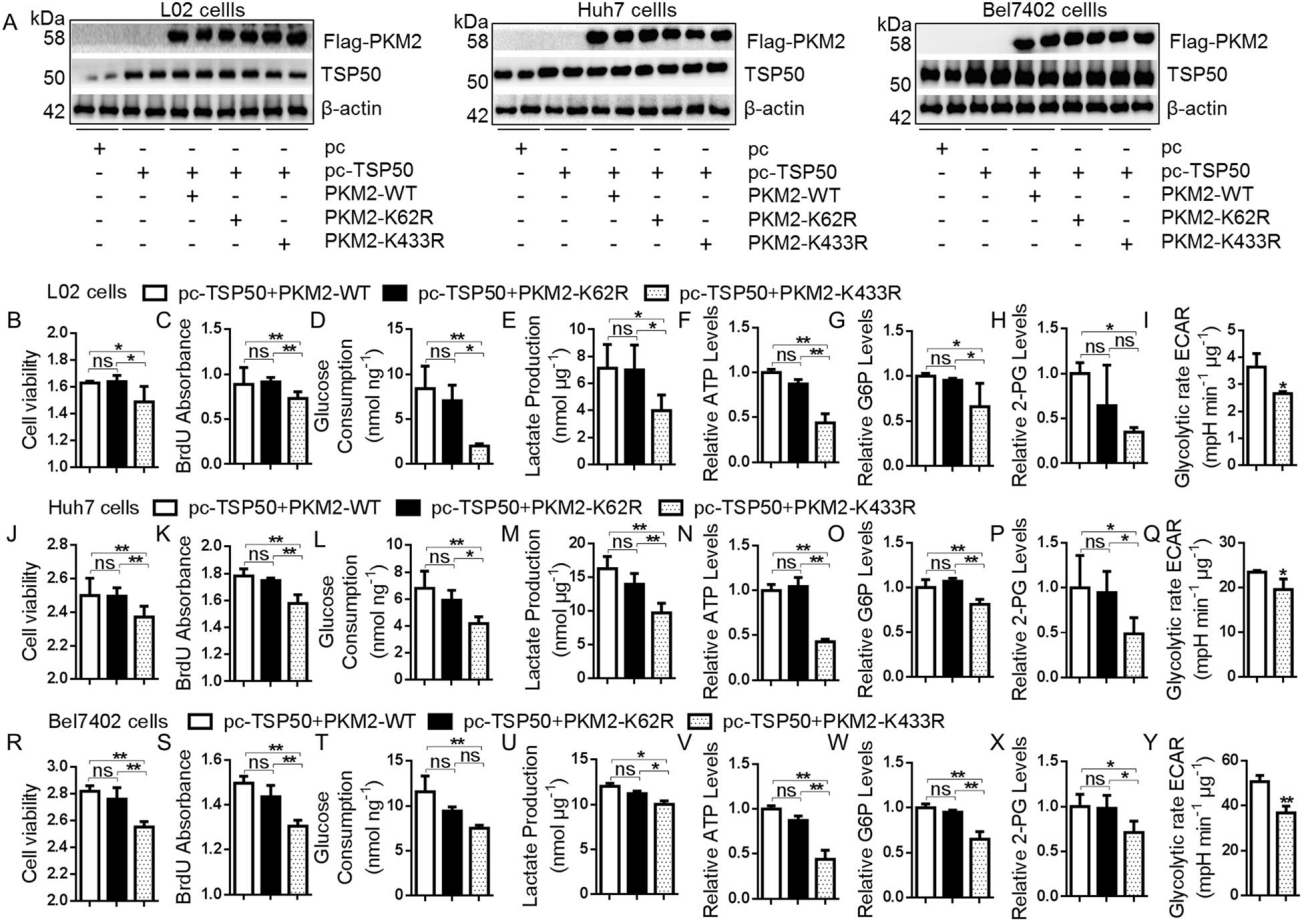
TSP50 mediates the cell proliferation and aerobic glycolysis changes partially dependent on PKM2 K433 acetylation. **A** The pcDNA3.1-TSP50 and Flag-PKM2 WT, Flag-PKM2 K62R or Flag-PKM2 K433R mutant plasmids were co-transfected into L02, Huh7 and Bel7402 cells, respectively. **B**–**Y** The cell viability, BrdU absorbance, glucose consumption, lactate production, ATP levels, G6P levels, 2-PG levels and ECAR levels were detected. The aerobic glycolysis values were normalized to protein levels. *N* = 3 biologically independent replicates. One-way ANOVA statistical analysis was used. Data were presented as means ± s.d. *P < 0.05, ** P < 0.01. ns, no significance.

Subsequently, the role of PKM2 K433 acetylation in TSP50-induced cell proliferation and aerobic glycolysis was verified. We co-transfected TSP50 with PKM2 K433R into L02 and HCC cells, and the TSP50 + PKM2 WT and TSP50 + PKM2 K62R were used as controls (Fig. 7A). After transfection for 48 h, MTT and BrdU results showed that the viability and proliferation level of cells in the K433R group were significantly lower than that in the PKM2 WT and PKM2 K62R groups (Fig. 7B, C, J, K, R–S). The levels of aerobic glycolysis were further detected, and the results showed that glucose consumption, lactate production, ATP, G6P and 2PG levels decreased significantly in K433R group (Fig. 7D–H, L–P, T–X). Furthermore, the ECAR levels in the TSP50 and PKM2 K433R expressing group were significantly lower than that in the control groups (Fig. 7I, Q and Y). Taken together, these results suggested that the regulation of TSP50 on cell proliferation through the glycolysis pathway depended on the acetylation level of PKM2 K433 site.

### PKM2 K433 acetylation is required for TSP50-induced tumor growth

**Fig. 8:**
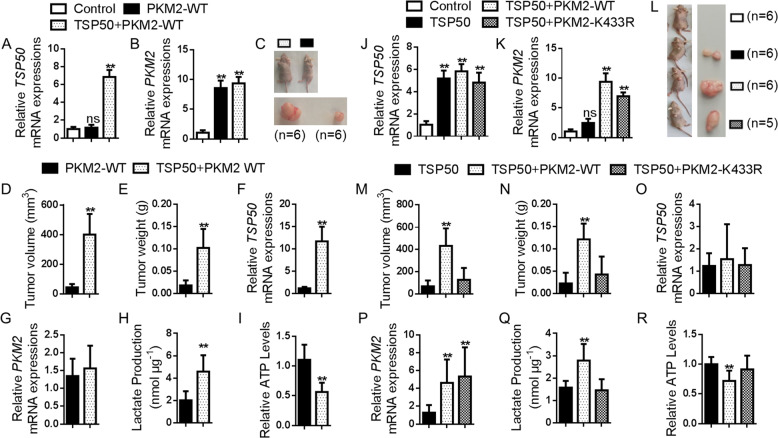
PKM2 K433 acetylation is required for TSP50-induced tumor growth. **A**–**C** Photography of xenograft tumor-bearing mice injected with different cell lines transfected. **D**, **E** Tumor volume and tumor weight for each group. **F**–**I** Expression of TSP50 and PKM2, lactate production and ATP levels were detected in tumor. **J**–**L** Photography of xenograft tumor-bearing mice injected with different cell lines transfected. **M**, **N** Tumor volume and tumor weight for each group. **O**–**R** Expression of TSP50 and PKM2, lactate production and ATP levels were detected in tumor. *n* = 6 in each group. All aerobic glycolysis values were normalized to protein levels. *N* = 3 biologically independent replicates. *t*-Test or one-way ANOVA statistical analysis was used. Data were presented as means ± s.d. *P < 0.05, **P < 0.01. ns, no significance.

To extend our studies into an animal model, we examined the role of PKM2 K433 acetylation in regulating the growth of TSP50-induced tumors in immune-deficient mice in vivo and assayed for tumor formation (*n* = 6/group). The TSP50 and PKM2 WT-stable-expressed L02 cells were prepared by lentivirus infection (Fig. 8A, B) which were used for female athymic nude mice dorsal subcutaneous injection. All immune-deficient mice in PKM2 WT and TSP50 + PKM2 WT groups emonstrated tumor formation by 4 weeks post-injection. Compared with the PKM2 WT group, the tumor volume and weight in TSP50 + PKM2 WT group were significantly increased (Fig. 8C–E). The relative expression of TSP50 and PKM2 was consistent with that in stably transfected cells (Fig. 8F, G). Meanwhile, higher lactate levels and lower ATP levels were also detected in TSP50 + PKM2 WT group (Fig. 8H, I).

Furthermore, we constructed a PKM2 K433R-stable expressed L02 cell line for nude mice subcutaneously injection (Fig. 8J, K). After 4 weeks in vivo tumour growth, the mice were sacrificed, and tumour growth was assessed. Compared with the NC group, TSP50 significantly promoted tumor formation. On the other hand, in TSP50-overexpressing cells, PKM2 K433R transfection group demonstrated a smaller tumor size (vs. PKM2 WT group as a control), accompanied by a dramatically reduced tumor weight (Fig. 8L–N). The relative expression of TSP50 and PKM2 was consistent with that in stably transfected cells (Fig. 8O, P). In addition, tumor tissues in the TSP50 + PKM2 WT group showed increased lactate production and reduced ATP levels (Fig. 8Q–R).

In summary, the results showed that TSP50 obviously enhanced PKM2 WT-cells growth, however, it has no effect on the PKM2 K433R-cells. These findings indicated that TSP50 played a key role in cell proliferation and tumor growth, at least in part, by regulating PKM2 K433 acetylation through aerobic glycolysis pathway.

## Discussion

Metabolic reprogramming is recognized as one of the hallmarks of cancer^51^. Even with sufficient oxygen, the energy is supplied mainly through glycolytic metabolic pathways in tumor cells, which is known as Warburg effect. Aerobic glycolysis signature enables tumor cells to satisfy anabolic and energetic needs for biological macromolecules production. This metabolic is involved in regulating the rapid proliferation and apoptosis resistance of tumor cells^38,52^. Many human cancers, including HCC, exhibit an aerobic glycolytic phenotype, which is often associated with tumor progression and poor clinical outcomes in cancer patients^4–6,53,54^. Therefore, tumor-specific aerobic glycolysis may be potential therapeutic target for HCC treatment. The pyruvate kinase M2 (PKM2) isoform, which is commonly upregulated in many human cancers, has been recently shown to play a crucial role in catalyzing aerobic glycolysis. The decrease of PKM2 activity significantly promotes the level of aerobic glycolysis^33,55^. High glycolysis flux is affected by the activation of oncogenes and mutations of tumor suppressor genes^52^. Nevertheless, the molecular regulatory mechanisms remain unclear.

Here, we demonstrate that oncogene TSP50 promotes the Warburg effect in HCC and reveal an underlying molecular mechanism: TSP50 maintains low PKM2 pyruvate kinase activity by increasing the acetylation level of PKM2 in cytoplasm. Through this regulation, TSP50 can promote the proliferation of HCC cells both in vivo and in vitro and may be a potential molecular target for HCC therapy.

TSP50 is abnormally highly expressed in various tumor tissues, but is absent in normal tissues^16–20^, suggesting a pathogenic role for TSP50 in cancers. TSP50 levels appear to have important clinical implications for cancer patients^18,23,56^, therefore, we evaluated TSP50 transcription levels via UALCAN database. Analysis results revealed that mRNA expression of TSP50 was significantly higher in TCGA-LIHC tissues than in adjacent normal tissues. GEPIA database analysis results showed that the high TSP50 expression group had a shorter RFS. Thus, TSP50 expression may serve as a potential diagnostic indicator in HCC and deserves further experimental validation.

In this study, an abnormally high expression of TSP50 was detected in HCC cells and TSP50 level was positively correlated with expression of glycolytic genes, supporting its relevance for the glycolytic phenotype of HCC cells. We further examined the regulation of TSP50 as a proto-oncogene on the Warburg effect, and we demonstrated that TSP50 knockdown impaired the aerobic glycolytic phenotype and survival in cultured Huh7 and Bel7402 cells. On the contrary, TSP50-overexpressed L02 cells showed an enhanced Warburg effect, which was reversed by a glycolysis inhibitor 2-DG. Meanwhile, the proliferation-promoting effect of TSP50 was partially attenuated by the addition of 2-DG. This suggested that TSP50 may promote both HCC initiation and maintenance at least in part by upregulating glycolytic metabolism.

We speculated that the effect of TSP50 on aerobic glycolysis may be mediated by some key factors such as Glut1, HK2 and PKM2. LC-MS/MS detection results showed that TSP50 might interact with PKM2, and we verified this results. Furthermore, TSP50 is dependent on PKM2 for its metabolic effects. Recent studies have confirmed that the low PK activity of PKM2 plays a key role in promoting the Warburg effect and tumor cell survival^31–33^. We indeed found that TSP50 maintained low PKM2 pyruvate kinase activity in TSP50-transfected L02 cells and this was also demonstrated by the enhanced pyruvate kinase activity of PKM2 in TSP50 knock-downed HCC cells. Studies have shown that PKM2 activity is regulated by metabolic intermediates^34^, phosphorylation modification^41,42,57,58^, acetylation modification^43,44^, O-GlcNAcylation modificatio^45^, protein-protein interaction^59,60^, transcriptional regulation and selective splicing^61^, and miRNAs^62,63^. So how does TSP50 decrease PKM2 pyruvate kinase activity? Our results showed that TSP50 promoted PKM2 K433 acetylation by increasing PKM2-bound KAT9 level, and decreasing SIRT2 level in the complex in cytoplasm. However, we cannot define whether TSP50 can directly acetylate PKM2 which needs further studied. Furthermore, PKM2 K433R mutation inhibited TSP50-induced tumor formation and aerobic glycolysis in immune-deficient mice in vivo.

It is known that high acetylation level of PKM2 can reduce its enzyme activity and promote aerobic glycolysis^43,44^, which is consistent with our findings in HCC cells. Studies on PKM2 K433 acetylation show that PKM2 is acetylated at K433 by p300 and function as a protein kinase for gene expression regulation in nucleus to promote cell proliferation^43^. A previous study also showed that the MCL treatment reduces the acetylation at K433, contributing to the tetramerization of PKM2 indirectly and inhibiting nuclear translocation in cells, which further suppressing the transcriptional regulation in tumorigenesis^50^. In the activated dendritic cells (DCs) induced by LPS, JNK promotes PKM2-K433ac, resulting in the decreased PKM2 enzymatic activity and high glycolysis level^49^. PKM2 also have many nonglycolytic functions. For example, PKM2 K433 acetylation is found to facilitate PKM2-DDB2 binding, thus affecting cell survival, which is consistent with UV irradiation^64^. However, in our study, we mainly analyzed the effect of TSP50-mediated PKM2 K433 acetylation on its pyruvate kinase activity in the cytoplasm. We found that PKM2 was acetylated at K433 by KAT9 and deacetylated by SIRT2. Interestingly, studies have shown that PKM2 lysines 305 is a direct SIRT2 deacetylation targets^46^. Recent studies have also suggested HDAC7 to deacetylate PKM2 at lysine 433^65^. We speculate that this may be regulated by TSP50. However, these inferences need further analysis to be confirmed. Additionally, we cannot clarify the role of TSP50 in affecting the binding ability of PKM2 to KAT9 or SIRT2 and the exact regulatory mechanism is unclear. Therefore, it is necessary to conduct further experiments to analyze this phenomenon. Furthermore, TSP50 may regulate tumor metabolism through a variety of pathways. Are these pathways acting independently or intersecting? Which is most effective? These problems also need to be further investigated.

In conclusion, TSP50 acetylates the PKM2 K433 site for low PKM2 pyruvate kinase activity to promote the Warburg effect, which is benefit for HCC cells survival, and we reveal a new regulatory mechanism of TSP50 in HCC progression. These results may provide new ideas for the treatment of human HCCs with TSP50 as the target.

## Materials and methods

### UALCAN database and GEPIA database analysis

The expression level of the TSP50 gene in HCC and normal tissues was analyzed in UALCAN (http://ualcan.path.uab.edu/). Meanwhile, the database was used to analyze the expression of TSP50 in various tumor sub-groups based on individual cancer stages, gender, age, and tumor grade. The correlation between TSP50 mRNA expression and survival of HCC patients was analyzed by the GEPIA database (http://gepia.cancer-pku.cn/). GEPIA searches for relationships between gene expression and patient prognosis, such as RFS, across a large collection of publicly available HCC RNA-seq datasets.

### Cell culture and plasmid transfection

L02 cells, Huh7 cells, Bel7402 cells and HEK-293T cells were obtained from the Chinese Academy of Sciences. Both L02 and Bel7402 cell lines used were pure. All cells were authenticated and tested for mycoplasma contamination. L02 cells were cultured in Roswell Park Memorial Institute 1640 medium supplemented with 20% fetal bovine serum (FBS). Huh7 cells and Bel7402 cells were cultured in RPMI 1640 medium with 10% FBS. The HEK-293T cells were cultured in H-DMEM medium supplemented with 10%FBS. All mediums were supplemented with 100 units/ml penicillin and 100 mg/ml streptomycin. Cells were maintained at 37 °C in incubator containing 5% CO_2_. When the cells confluence reached 80%, 200 µl RPMI 1640 serum-free medium was mixed with 5 µl of X-tremeGENE HP and 2 µg plasmid for transfection. The primers for plasmid construction were shown in Supplementary Table 1.

### qRT-PCR detection and Western blot

Total RNA was isolated using Trizol reagent and cDNAs were synthesized by a RT-PCR Kit and specific primers were designed for qRT-PCR detection (Supplementary Table 2). The detection conditions and procedures were set according to instructions provided in Sybr green kit. The proteins were extracted with RIPA buffer and isolated by SDS-PAGE and transferred to PVDF membrane. After blocking with 5% skim milk, the membrane was then incubated using the primary antibodies with suggested dilutions. The immunoblotting was detected by ECL luminous fluid.

### MTT and BrdU assay

The cell viability or proliferation was detected using MTT or BrdU ELISA kit. The cells were seeded in 96-well cell plates (2 × 10^3^ cells, 100 μL/well), and three duplicate wells were set for each group. 48 h after transfection, 20 μL (5 mg/mL) of MTT solution was added to each well, and cells were continuously cultured in the incubator for 4–6 h. Finally, labeling was stopped using DMSO and MTT uptake was measured according to the protocol of the manufacturer. For BrdU analysis, the medium was incubated with a 10 μM BrdU labeling solution for 6 h. 48 h after transfection, BrdU uptake was measured according to the manufacturer’s protocol.

### Analysis of glucose metabolism

The transfected cells were seeded into 96-well plates, and the medium was replaced with fresh complete medium after 6 h. The medium was collected to measure the concentration of glucose consumption and lactate production after additional 48 h of incubation. Glucose and lactate levels were measured using glucose assay kit and lactate detection kit respectively according to the manufacturer’s instructions. All values were normalized to protein levels.

### Glycolysis level detection

Levels of lactate dehydrogenase (LD) was analyzed by a lactate dehydrogenase assay kit according to the manufacturer’s protocol. The cells were treated with 1.5 μM oligomycin, luminometer and ATP detection kit were used to detect intracellular ATP levels in cell lysates. Levels of glucose-6-phosphate and 2-phosphoglycerate were analyzed using G6P Assay Kit and 2-PG ELISA kits respectively according to the manufacturer’s protocol. All values were normalized to protein levels.

### Oxygen consumption rate and extracellular acidification rate detection

Oxygen consumption rate (OCR) and extracellular acidification rate (ECAR) were detected using the Seahorse XFp Extracellular Flux Analyzer (Seahorse Bioscience, North Billerica, MA). The cells (1 × 10^4^) were seeded into a Seahorse XFp cell culture microplate. For OCR, oligomycin, p-trifluoromethoxy carbonyl cyanide phenylhydrazone (FCCP), and rotenone were added at the indicated time points. When detecting ECAR, glucose, oligomycin, and 2-deoxyglucose (2-DG) were added in sequence at the indicated time points. Finally, data were analyzed using the Seahorse XFp Wave software. Both ECAR and OCR measurements were normalized to protein levels.

### LC–MS/MS analysis of interacting proteins

The total protein of Huh7 cells was separated by polyacrylamide gel electrophoresis (SDS-PAGE) and the protein bands (50 kDa) were excised after Coomassie brilliant blue staining. After decolorization, dehydration, protein reduction, protein alkylation, enzymolysis and extraction, the processed peptides were analyzed using a liquid chromatography masss pectrometry/mass spectrometry (LC–MS/MS) method to obtain the proteins specifically binding to TSP50 in Huh7 cells and characterization of the peptides was carried out by Sangon Biotech.

### Co-immunoprecipitation (Co-IP)

The Pierce Crosslink Immunoprecipitation kit from Thermo Scientific was used for Co-immunoprecipitation experiments detection according to the manufacturer’s protocol.

### GST pull-down assay

The recombinant pGEX-4T-1-GST-TSP50 plasmid was transformed into BL21 competent bacteria, and the monoclonal colonies were selected and cultured in shaker at 37 °C until OD600 = 0.6~0.8. After 6 h of induction with 1 mM IPTG, the precipitated bacteria were collected after centrifugation. Finally, GST-tag Protein Purification Kit was used to purify GST-TSP50 protein according to the manufacturer’s instructions.

The total protein of Huh7 cells or Bel7402 cells was extracted using RIPA lysate. Then, the target protein was mixed with GST pull-down protein binding buffer, well-balanced 50% gel suspension and bait protein rolling at 4 °C overnight. After centrifugation, the supernatant was removed, and GST pull-down binding buffer was added to resuspend the gel to sufficiently wash off unbound protein. Finally, GST pull-down elution buffer was added to gel and incubated for 10 min. After centrifugation at 4 °C 1000 *g* for 2 min, the supernatant was collected for Western blot detection.

### Immunofluorescence detection

Cells were seeded on sterile coverslips in six-well culture plates. After 24 h, the cells were fixed in 4% paraformaldehyde and washed three times with PBS. Then, 0.1% Triton X-100 was used to permeate cells at room temperature for 5 min, and non-specific sites were then blocked with 5% bovine serum albumin for 30 min. Thereafter, primary antibodies were flooded over the cells, and the culture was incubated at 4 °C overnight. Furthermore, the cells were incubated with FITC or HRP-conjugated secondary antibody for 1 h in the dark at room temperature. Nuclei were stained with DAPI for 5 min at room temperature. Finally, fluorescence analysis was performed using an Olympus BX50 fluorescence microscope (Olympus, Tokyo, Japan).

### PKM2 oligomerization assay

An equal amount of whole cell lysate was collected and cross-linked with 0.025% glutaraldehyde at 37 °C for 3 min, and then the reaction was terminated with Tris·HCl (pH 8.0, 50 mM). Finally, the processed samples were boiled and analyzed with Western blotting using the indicated antibodies.

### PK activity assay

The cells were collected into the centrifuge tube and the supernatant was discarded after centrifugation. The cells were broken by ultrasound (ice bath, power 20% or 200 W, ultrasound for 3 s, 10 s intervals, repeated 30 times) for PK extraction. After centrifugation at 8000 *g*, 4 °C for 10 min, the supernatant was taken for further detection. The working solution and the cell lysate were mixed and incubated, and the PK activity was measured using a colorimetric-based Pyruvate kinase (PK) activity detection kit according to the manufacturer’s protocol.

### Lentiviral packaging and tumorigenicity studies

HEK-293T cells were cultured in H-DMEM medium supplemented with 10%FBS. Human TSP50, PKM2 or PKM2 K433R gene was cloned into Plvx-AcGFP-N1 to construct vector plasmids. Vector plasmids, packaging genome plasmids pMD2G and pSPAX2 were co-transfected into 293 T cells. After 48 h, the supernatants of the culture medium were collected and filtered by 0.45 μm filter. The virus was concentrated by ultracentrifugation and stored at −80 °C.

All animal studies were conducted with approval from the Animal Research Ethics Committee of Northeast Normal University (NENU/IACUC, AP20191225) of China and performed in accordance with established guidelines. Female BALB/C nude mice (6 weeks old) were purchased from Charles River Animal Company of China, and kept in a non-pathogenic condition. The mice were randomly blindly assigned to experimental groups (*n* = 6 in each group). Lentivirus-infected L02 cells were digested with trypsin after 48 h, washed and resuspended in PBS at 5 × 10^7^ cells/ml. Then, 200 μl of treated cells were injected subcutaneously into female nude mice. After 4 weeks, the mice were sacrificed with cervical dislocation, and the xenografts were resected and weighed. The volume of xenograft was measured with vernier caliper and calculated by the following equation: V = L × W^2^ × 0.5 (L, length; W, width of the xenografts).

### Statistical analysis

IBM SPSS Statistics was used for statistical analysis. The data were presented as mean ±S.D. All data were from three independent experiments. *P*-values were calculated from Two-sided Student’s *t*-test test or one-way ANOVA, *P < 0.05 and **P < 0.01 were displayed as statistical significance, ns, no significant.

## Supplementary information

Figure S1

Figure S2

Figure S3

Table 1

Table 2

Supplementary figure and table legends
