# Supplementary figures and images for: TSP50 promotes the Warburg effect and hepatocyte proliferation via regulating PKM2 acetylation
Source: Cell Death Dis. 2021 May 20;12(6):517. doi: 10.1038/s41419-021-03782-w (PMC8138007; doi:10.1038/s41419-021-03782-w)

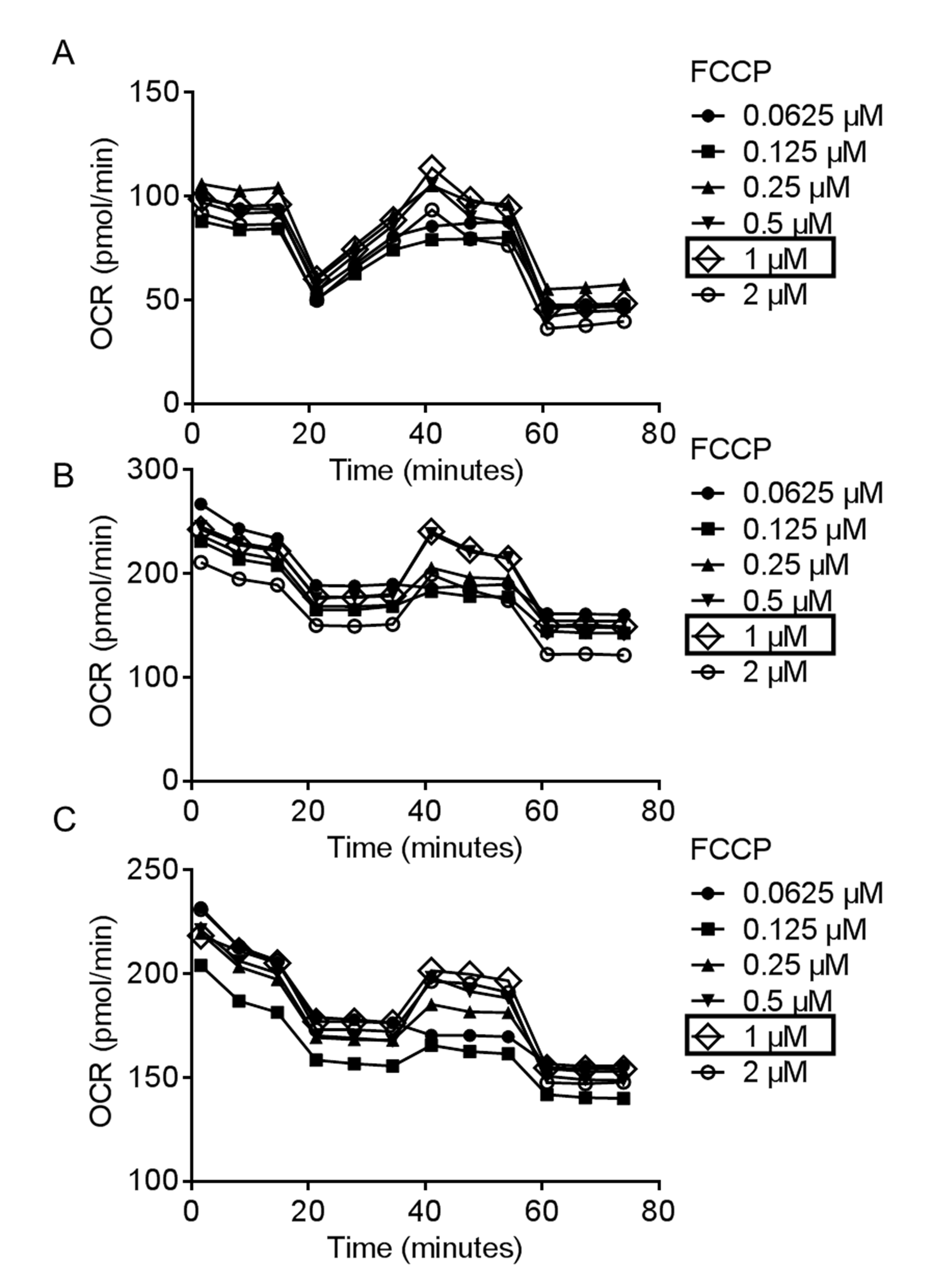

Supplement: Supplementary file 1 — Figure S1 [file 41419_2021_3782_MOESM1_ESM.tif]

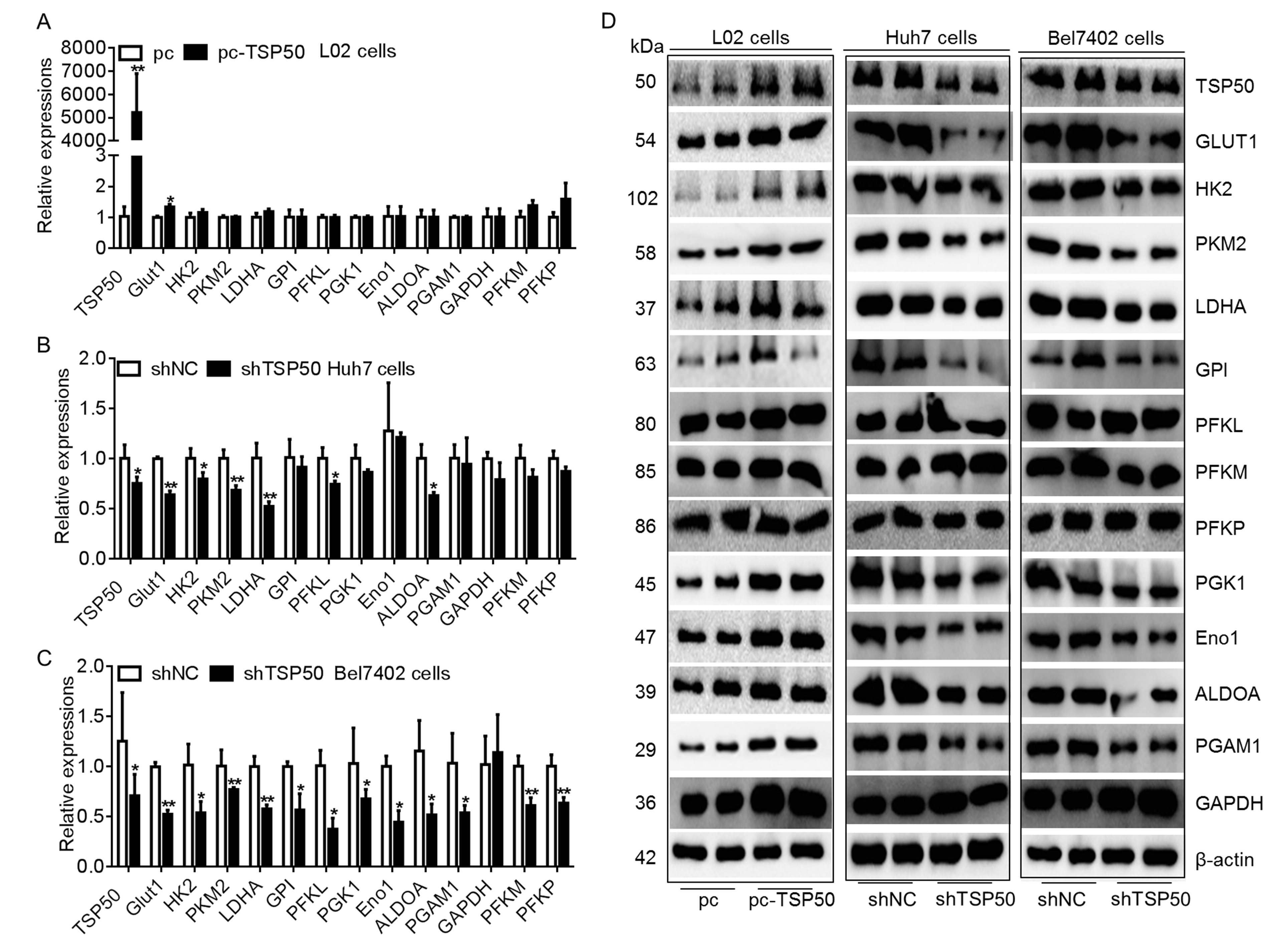

Supplement: Supplementary file 2 — Figure S2 [file 41419_2021_3782_MOESM2_ESM.tif]

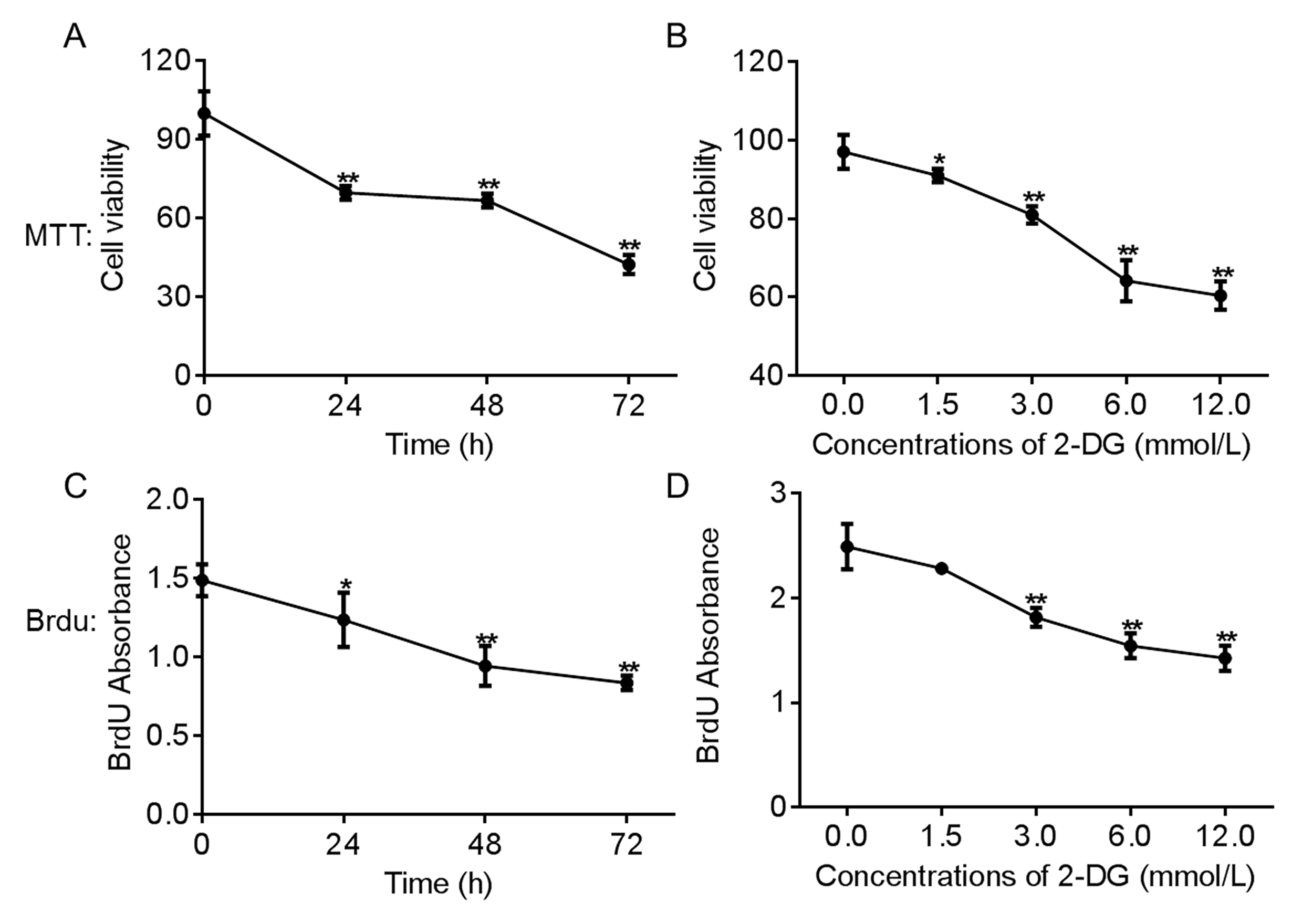

Supplement: Supplementary file 3 — Figure S3 [file 41419_2021_3782_MOESM3_ESM.tif]
