# Supplementary material for: TSP50 promotes the Warburg effect and hepatocyte proliferation via regulating PKM2 acetylation
Source: Cell Death Dis. 2021 May 20;12(6):517. doi: 10.1038/s41419-021-03782-w (PMC8138007; doi:10.1038/s41419-021-03782-w)
Supplement: Supplementary file 4 — Table 1 [file 41419_2021_3782_MOESM4_ESM.docx]

Table 1 Primers for PKM2 mutation vector construction

| *Symbol* | *Primer* | *Primer Sequence (5′–3′)* |
| --- | --- | --- |
| PKM2 K62R | F-Primer  R-Primer | CCCGATCAGTGGAGACGTTGAGGGAGATGATTAAGTC  GACTTAATCATCTCCCTCAACGTCTCCACTGATCGGG |
| PKM2 K305R | F-Primer  R-Primer | GGCATTGAGATTCCTGCAGAGAGGGTCTTCCTTGCTC  GAGCAAGGAAGACCCTCTCTGCAGGAATCTCAATGCC |
| PKM2 K433R  PKM2 K433Q | F-Primer  R-Primer  F-Primer  R-Primer | CCATAATCGTCCTCACCAGGTCTGGCAGGTCTGCTCA  TGAGCAGACCTGCCAGACCTGGTGAGGACGATTATGG  CCATAATCGTCCTCACCCAGTCTGGCAGGTCTGCTCA  TGAGCAGACCTGCCAGACTGGGTGAGGACGATTATGG |
