# Supplementary material for: TSP50 promotes the Warburg effect and hepatocyte proliferation via regulating PKM2 acetylation
Source: Cell Death Dis. 2021 May 20;12(6):517. doi: 10.1038/s41419-021-03782-w (PMC8138007; doi:10.1038/s41419-021-03782-w)
Supplement: Supplementary file 5 — Table 2 [file 41419_2021_3782_MOESM5_ESM.doc]

Table 2 Primer sequences for qRT-PCR detection

| *Symbol* | *Primer* | *Primer Sequence (5′–3′)* |
| --- | --- | --- |
| TSP50 | F-Primer  R-Primer | ACAGGGAGGAGTTCTGCTATGAGATAAC  AAAGATGGGTGGGGCCTCGCTCTTCTTG |
| Glut1 | F-Primer  R-Primer | ATTGGCTCCGGTATCGTCAAC  GCTCAGATAGGACATCCAGGGTA |
| HK2  PKM2  LDHA  GPI  PFKL  PGK1  Eno1  ALDOA  PGAM1  GAPDH  PFKM  PFKP  β-actin | F-Primer  R-Primer  F-Primer  R-Primer  F-Primer  R-Primer  F-Primer  R-Primer  F-Primer  R-Primer  F-Primer  R-Primer  F-Primer  R-Primer  F-Primer  R-Primer  F-Primer  R-Primer  F-Primer  R-Primer  F-Primer  R-Primer  F-Primer  R-Primer  F-Primer  R-Primer | TTGACCAGGAGATTGACATGGG  CAACCGCATCAGGACCTCA  GTGCGAGCCTCAAGTCACTCCACA  TATAAGAAGCCTCCACGCTGCCCA  TTGACCTACGTGGCTTGGAAG  GGTAACGGAATCGGGCTGAAT  CAAGGACCGCTTCAACCACTT  CCAGGATGGGTGTGTTTGACC  GCTGGGCGGCACTATCATT  TCAGGTGCGAGTAGGTCCG  GAACAAGGTTAAAGCCGAGCC  GTGGCAGATTGACTCCTACCA  GCCGTGAACGAGAAGTCCTG  ACGCCTGAAGAGACTCGGT  ATGCCCTACCAATATCCAGCA  GCTCCCAGTGGACTCATCTG  GTGCAGAAGAGAGCGATCCG  CGGTTAGACCCCCATAGTGC  GGAGCGAGATCCCTCCAAAAT  GGCTGTTGTCATACTTCTCATGG  GGTGCCCGTGTCTTCTTTGT  AAGCATCATCGAAACGCTCTC  GACCTTCGTTCTGGAGGTGAT  CACGGTTCTCCGAGAGTTTG  CGTGCGTGACATTAAGGAGAAG  GGAAGGAAGGCTGGAAGAGTG |
