# Supplementary material for: TSP50 promotes the Warburg effect and hepatocyte proliferation via regulating PKM2 acetylation
Source: Cell Death Dis. 2021 May 20;12(6):517. doi: 10.1038/s41419-021-03782-w (PMC8138007; doi:10.1038/s41419-021-03782-w)
Supplement: Supplementary file 6 — Supplementary figure and table legends [file 41419_2021_3782_MOESM6_ESM.docx]

**Fig.S1.** **Screening of FCCP concentration for OCR detection.** (A) FCCP concentration screening in L02 cells. (B) FCCP concentration screening in Huh7 cells. (C) FCCP concentration screening in Bel7402 cells.

**Fig.S2. TSP50 affects aerobic glycolysis-related genes expression.** (A-C) The qRT-PCR analysis results in TSP50-overexpressed L02 cells and TSP50-knockdown Huh7 and Bel7402 cells. (D) Expression of proteins were detected by Western blot in TSP50-overexpressed L02 cells and TSP50-knockdown Huh7 and Bel7402 cells. N=3 biologically independent replicates. T-test statistical analysis was used. Data were presented as means ± s.d. **P<0.05*, *** P<0.01*.

**Fig.S3. Screening of action time and concentration of 2-DG.** (A-D) The action time and concentration were analyzed by MTT and BrdU assay.

**Table 1. Primers for PKM2 mutation vector construction.**

**Table 2. Primer sequences for qRT-PCR detection.**
